# Comparison Study of the Bio-Plex and Meso Scale Multiplexed SARS-CoV-2 Serology Assays Reveals Evidence of Diminished Host Antibody Responses to SARS-CoV-2 after Monoclonal Antibody Treatment

**DOI:** 10.20411/pai.v9i2.715

**Published:** 2024-08-15

**Authors:** Urvi M. Parikh, Amy L. Heaps, Daniela Moisi, Kelley C. Gordon, John W. Mellors, Manish C. Choudhary, Rinki Deo, Carlee Moser, Paul Klekotka, Alan L. Landay, Judith S. Currier, Joseph J. Eron, Kara W. Chew, Davey M. Smith, Jonathan Z. Li, Scott F. Sieg, Team ACTIV-2/A5401 Study

**Affiliations:** 1 University of Pittsburgh School of Medicine, Pittsburgh, PA; 2 Case Western Reserve University, Cleveland, OH; 3 Department of Medicine, Division of Infectious Diseases, Brigham and Women's Hospital, Harvard Medical School, Boston, MA; 4 Department of Biostatistics, Harvard T.H. Chan School of Public Health, Boston, MA; 5 Eli Lilly and Company, San Diego, CA; 6 Department of Internal Medicine, Division of Geriatrics and Palliative Medicine, RUSH Medical College, Chicago, IL; 7 Department of Medicine, David Geffen School of Medicine at University of California Los Angeles, Los Angeles, CA; 8 Department of Medicine, University of North Carolina at Chapel Hill, Chapel Hill, NC; 9 Department of Medicine, University of California, San Diego, CA; 10 Case Western Reserve University and University Hospitals Cleveland, Cleveland, OH; 11 ACTIV-2/A5401 Study Team: David Smith, Kara Chew, Eric Daar, David Wohl, Judith Currier, Joseph Eron, Arzhang Cyrus Javan, Michael Hughes, Carlee Moser, Justin Ritz, Mark Giganti, Lara Hosey, Jhoanna Roa, Nilam Patel, Kelly Colsh, Irene Rwakazina, Justine Beck, Scott Sieg, Jonathan Li, Courtney Fletcher, William Fischer, Teresa Evering, Rachel Bender Ignacio, Sandra Cardoso, Katya Corado, Prasanna Jagannathan, Nikolaus Jilg, Alan Perelson, Sandy Pillay, Cynthia Riviere, Upinder Singh, Babafemi Taiwo, Joan Gottesman, Matthew Newell, Susan Pedersen, Joan Dragavon, Cheryl Jennings, Brian Greenfelder, William Murtaugh, Jan Kosmyna, Morgan Gapara, Akbar Shahkolahi

**Keywords:** SARS-CoV-2, COVID, COVID-19, Bio-Plex, Meso Scale Discovery, monoclonal antibodies, bamlanivimab

## Abstract

**Background:**

Assessing the breadth and duration of antigen-specific binding antibodies provides valuable information for evaluating interventions to treat or prevent SARS-CoV-2 infection. Multiplex immunoassays are a convenient method for rapid measurement of antibody responses but can sometimes provide discordant results, and antibody positive percent agreement for COVID-19 diagnosis can vary depending on assay type, disease severity, and population sampled. Therefore, we compared two assays marked for research applications, MSD and Bio-Plex Pro, to evaluate qualitative interpretation of serostatus and quantitative detection of antibodies of varying isotypes (IgG, IgM, and IgA) against receptor binding domain (RBD) and nucleocapsid (N) antigens.

**Methods:**

Specimens from ACTIV-2/A5401, a placebo-controlled clinical trial of the SARSCoV-2 monoclonal antibody (mAb) bamlanivimab to prevent COVID-19 disease progression, were used to evaluate the concordance of the Bio-Rad Bio-Plex Pro Human SARS-CoV-2 Serology Assay and the Meso Scale Discovery (MSD) V-PLEX COVID-19 Panel 1 serology assay in detecting and quantifying IgG, IgA, and IgM binding anti-SARS-CoV-2 antibody responses against the RBD and N antigens. Data were disaggregated by study arm, bamlanivimab dose, days post-enrollment, and presence of emerging resistance.

**Results:**

We observed 90.5% (412 of 455 tests) concordance for anti-RBD IgG and 87% (396 of 455) concordance for anti-N IgG in classifying samples as negative or positive based on assay-defined cutoffs. Antibody levels converted to the WHO standard BAU/mL were significantly correlated for all isotypes (IgG, IgM, and IgA) and SARS-CoV-2 antigen targets (RBD and N) tested that were common between the two assays (Spearman r 0.65 to 0.92, *P* < 0.0001). Both assays uncovered evidence of diminished host-derived IgG immune responses in participants treated with bamlanivimab compared to placebo. Assessment of immune responses in the four individuals treated with the 700 mg of bamlanivimab with emerging mAb resistance demonstrated a stronger anti-N IgG response (MSD) at day 28 (median 2.18 log BAU/mL) compared to participants treated with bamlanivimab who did not develop resistance (median 1.55 log BAU/mL).

**Conclusions:**

These data demonstrate the utility in using multiplex immunoassays for characterizing the immune responses with and without treatment in a study population and provide evidence that monoclonal antibody treatment in acute COVID-19 may have a modest negative impact on development of host IgG responses.

## INTRODUCTION

Measurement of antigen-specific antibodies in blood can provide insight into the magnitude, breadth, and duration of antibody responses that develop from natural infection or vaccination [1, 2]. Moreover, assessment of binding antibodies in clinical trials, such as those recently employed to assess interventions in SARS-CoV-2 infections, can be informative for understanding virologic and clinical outcomes [3]. United States Food and Drug Administration (FDA) emergency use authorized serological assays that qualitatively classify the presence of antibodies have been used diagnostically since the beginning of the COVID-19 pandemic, and a variety of research-use only approaches have also been developed to quantitatively measure SARS-CoV-2 antibodies in biological fluids [4].

Multiplex technology has long been used to measure cytokines and chemokines in biological fluids, and new assays were quickly adapted for SARS-CoV-2 to provide a convenient approach to assess binding antibodies of different isotypes and with different antigen specificities in a small volume of fluid. A well-defined multiplex approach includes the Meso Scale Discovery (MSD) platform that utilizes specific SARS-CoV-2 antigens spotted onto plates and anti-human antibodies conjugated with MSD SULFO-TAG™ electro-chemiluminescent detection to measure serum antibodies [5, 6]. MSD routinely updates its panels to include antigens from recently circulating variants through omicron sub-lineage XBB.1.5 to date with reference standards that are calibrated against the WHO International Standard; however, manufacturer qualitative cutoffs are not provided for all isotypes and antigens since the assay is not designed to be used diagnostically. This platform has played a major role in vaccine studies and is well-described in the literature [7–16].

A platform with similar capabilities is offered by Bio-Rad. The Bio-Plex Pro SARS-CoV-2 assay uses Luminex xMAP bead-based multiplex immunoassay technology to simultaneously detect antibody (IgG, IgA, or IgM) against the SARS-CoV-2 N, RBD, Spike 1 (S1), and Spike 2 (S2) antigens with fluorescence detection using biotin-streptavidin/phycoerythrin on a Bio-Plex 200 system. Unlike MSD, the Bio-Plex antigens are limited to the original Wuhan strain, with a developer kit available for researchers to generate their own assays with updated antigens. The addition of a standard curve to the test plate allows quantitation of antibody levels in arbitrary units per milliliter. The Bio-Plex 200 system is a relatively newer platform intended for research use, whereas the BioPlex 2200 ELISA/Platelia has been more widely used for clinical diagnosis in several COVID-19 vaccine and convalescent plasma studies [17–19].

It is not uncommon for different antibody assays to provide discordant results, and antibody positive percent agreement for COVID-19 diagnosis has varied depending on assay type, disease severity, and population sampled [19, 20]. Some investigators have suggested using two assays to validate results [21, 22]. Thus, we compared two assays marked for research applications, MSD and Bio-Plex Pro, to evaluate qualitative interpretation of serostatus and quantitative detection of antibodies of varying isotypes (IgG, IgM, and IgA) against receptor binding domain (RBD) and nucleocapsid (N) antigens. We first validated both assays using samples from a characterized repository, then investigated the concordance of these assays using specimens from ACTIV-2/A5401, a placebo-controlled clinical trial of the SARS-CoV-2 monoclonal antibody (mAb) bamlanivimab to prevent COVID-19 disease progression. We used both assays to analyze anti-N IgG activity in samples with and without bamlanivimab resistance in the mAb and placebo arms.

## METHODS

### Samples

Known SARS-CoV-2 antibody positive specimens for the validation studies were obtained from a SARS-CoV-2 specimen repository (Vitalant Research Institute). Pre-COVID sera collected prior to 2019 were blinded remnant specimens from HIV prevention clinical studies where participants without HIV had consented to their samples being used for future research. No identifying information was available for these samples. The ACTIV-2/A5401 study (NCT04518410) was a multicenter phase 2, randomized, placebo-controlled trial evaluating the efficacy of 700 and 7,000 mg of the mAb bamlanivimab to prevent COVID-19 disease progression in non-hospitalized symptomatic adults with documented SARS-CoV-2 infection enrolled within 10 days of symptom onset. The protocol was approved by a central institutional review board (IRB), Advarra (Pro00045266), with additional local IRB review and approval as required by participating sites. The dates of enrollment of the first and last participants for the bamlanivimab studies were August 19, 2020 and November 15, 2020. All participants provided signed, written informed consent and documentation was per site standard operating procedures. Half of participants were female (52.1%), and participant demographics were balanced between arms as previously reported [23]. Sera for this study was collected at baseline (pre-treatment), study day 28, and study week 12.

### Quantitative Bio-Rad Bio-Plex Pro Human SARS-CoV-2 Serology Assay

IgA, IgG, and IgM antibody titers against SARS-CoV-2 N, RBD, S1, and S2 antigens were measured using the multiplex magnetic bead-based Bio-Rad Bio-Plex Pro Human SARS-CoV-2 serology assays with the addition of a standard curve generated with the VIROTROL SARS-CoV-2 serological control (IgG) or assay positive controls (IgA or IgM) [24, 25]. Briefly, serum specimens were diluted at a range of 1:100 to 1:1,000 in sample diluent and incubated with SARS-CoV-2 N/RBD/S1/S2 4-plex antigen coupled beads for 30 minutes at ambient temperature, shaking at 850 rpm, then washed 3 times. The beads were then incubated with the kit-provided detection antibody for 30 minutes at ambient temperature, shaking at 850 rpm, and washed 3 times again. Using streptavidin-phycoerythrin as the fluorescent reporter, the assay was read on the Bio-Rad Bio-Plex 200 System. Results were interpreted using the Bio-Plex Manager Software, v6 and are reported as Units/mL (U/mL) for IgG or ng/mL for IgA and IgM measured against each standard curve. Threshold values to determine serostatus were provided by Bio-Rad for all antigens and isotypes and were based on analyses of 278 (IgG) or 282 (IgA/M) SARS-CoV-2 negative samples. The manufacturer-defined Bio-Plex cutoff for seropositivity was >450 mean fluorescence intensity units (MFI) for N, >250 MFI for RBD and S1, and >750 MFI for S2. Following manufacturer recommendations, thresholds were determined in-house for samples diluted greater than 100 by testing 22 samples obtained prior to 2019 at each reported dilution. A manufacturer supplied conversion factor generated using the National Institute for Biological Standards and Control (NIBSC) 20/136 reference standard was applied to translate units per milliliter to the World Health Organization (WHO) standard Binding Antibody Units (BAU)/mL [26].

### Meso Scale Discovery V-PLEX COVID-19 Plate 1 Serology Assay

SARS-CoV-2 antigen specific IgG, IgA, or IgM antibody levels in participant serum samples were quantitatively measured using Meso Scale Discovery (MSD) V-PLEX COVID-19 Plate 1 serology assay. The assays were run according to the kit package insert for serum samples. Briefly, the plate was blocked by incubation with the Blocker A solution for 30 minutes at room temperature with shaking at ~750 rpm. After 3 washes, the diluted serum samples, calibrators, and controls were added to the plate and incubated for 2 hours with shaking at room temperature. After a second round of washes, the detection antibody solution was added to the plate, followed by a 1-hour incubation. The plate was then washed, read buffer was added, and the plate was read on the MESO QuickPlex SQ 120MM Reader, which measures the light emitted from the MSD SULFO-TAG, and analyzed by the discovery Workbench software. Threshold values to determine serostatus were provided by MSD for anti-S, anti-RBD, and anti-N IgG antibodies and were based on analyses of 200 pre-2019 and 214 COVID+ (PCR-confirmed) COVID-19 patients. The manufacturer-defined MSD cutoff for seropositivity was >538 AU/mL (RBD), >1,960 AU/mL (S), and >5,000 AU/mL (N). NTD thresholds were not available. A manufacturer supplied conversion factor was applied to translate units per milliliter to the WHO standard BAU/mL [26].

### Statistical Analysis

Sensitivity and specificity were calculated using the proportion of true positive cases against the total number tested and the proportion of true negative cases against the total number tested, respectively; categorization of true positives was based on data provided by Vitalant Research Institute (Ortho-Clinical Diagnostics™ VITROS™ Immunodiagnostic Products Anti-SARS-CoV-2 IgG assay) that accompanied the repository samples, while all samples collected prior to 2019 were expected to be true negatives. For the qualitative analysis, manufacturer-defined or in-house determined mean fluorescence intensity (MFI) (Bio-Plex) or arbitrary units per milliliter (AU/mL) (MSD) cutoffs were used to define seropositivity for each isotype. Quantitative antibody correlation analysis was performed using the nonparametric Spearman Rank Correlation function in GraphPad Prism, version 10.1.2 (GraphPad Software, LLC). To compare median log BAU/mL between different arms over day of collection, the Mann-Whitney test for a 2-group non-parametric comparison was used to calculate statistical significance.

## RESULTS

### Validation of the Bio-Plex and Meso Scale Platforms Using Characterized Standards

Of 21 known IgG-positive SARS-CoV-2 samples, all 21 (100%) were IgG positive for anti-RBD (Bio-Plex and MSD) and anti-N (Bio-Plex), while MSD had two false IgG negatives for anti-N (19 of 21; 90.5%). Both MSD false negatives had values less than 2-fold lower than the qualitative cutoff of 5,000 AU/mL for anti-N IgG positivity (2,658 AU/mL and 2,617 AU/mL, respectively). All 10 pre-COVID specimens tested IgG negative for anti-RBD (Bio-Plex and MSD) and anti-N (MSD), while Bio-Plex had one false IgG positive for anti-N. The false positive sample was tested in triplicate and had values above 7-fold of the qualitative cutoff of 250 MFI for anti-N IgG positivity (3,121 ± 450 MFI) (Table 1).

**Table 1.** Sensitivity and Specificity of Anti-RBD and Anti-N IgG Using the Bio-Plex and Meso Scale Assays

Assay Platform	Isotype, Antigen	Source^a^	Sensitivity	Specificity
Bio-Rad Bio-Plex Pro Human SARS-CoV-2	IgG, anti-RBD	Manufacturer	100%	96%
		Validation	100%	100%
	IgG, anti-N	Manufacturer	100%	99%
		Validation	100%	90%
Meso Scale Discovery V-PLEX COVID-19	IgG, anti-RBD	Manufacturer	98.3%	98.5%
		Validation	100%	100%
	IgG, anti-N	Manufacturer	93.8%	100%
		Validation	90.5%	100%

a For Bio-Plex, manufacturer sensitivity and specificity was obtained from the Bio-Plex Pro Human IgG SARS-CoV-2 Serology Assays Product Data Sheet Version A, clinical specificity was determined using 278 specimens collection prior to December 2019, and clinical sensitivity was determined using 65 serum and plasma samples confirmed to be human IgG anti-SARS-CoV-2 positive. For Meso Scale, receiver operating characteristic curve (ROC) was used to determine sensitivity and specificity from 200 serum samples collected from healthy adults before 2019 and 214 PCR-confirmed COVID-19 positive individuals collected more than 15 days after diagnosis, as reported in the Meso Scale package insert.

### Qualitative Assessment of the Bio-Plex and Meso Scale Platforms

Total IgG antibody levels were assayed in sera by both the Bio-Plex and MSD platforms for 455 serum samples from 304 participants who received 700 or 7,000 mg bamlanivimab or placebo in the A5401 study. Bio-Plex and MSD were 90.5% concordant (412 of 455 tests) for anti-RBD IgG in classifying samples as negative or positive by both assays. Forty samples negative for anti-RBD IgG by MSD were positive by Bio-Plex. Of these 40, almost half (19 samples; 48%) were 3- to 10-fold above the Bio-Plex MFI cutoff of 250 defining IgG positivity, and 6 (15%) were >10-fold above the MFI cutoff. Conversely, 3 samples negative for anti-RBD IgG by Bio-Plex were positive by MSD. Two of the 3 samples were close to the anti-RBD MSD cutoff of 538 AU/mL for IgG positivity, while one sample was 13-fold above the cutoff.

Similar trends were seen for detection of anti-N IgG by both assays. The rate of concordance was 87%, with discordance of 12.3% for MSD negative samples testing positive by Bio-Plex for anti-N IgG, and 0.7% for Bio-Plex negative samples testing positive by MSD for anti-N IgG (Table 2).

**Table 2.** Qualitative Detection of Anti-RBD and Anti-N IgG Using the Bio-Plex and Meso Scale Assays

IgG	Assay Result	Bio-Plex Negative	Bio-Plex Positive	Concordant^a^	Discordant MSD NEG Bio-Plex POS	Discordant MSD POS Bio-Plex NEG
All Samples (N = 455)						
Anti-RBD	MSD Negative	212	40	412 (90.5%)	40 (8.8%)	3 (0.7%)
	MSD Positive	3	200			
Anti-N	MSD Negative	233	56	396 (87.0%)	56 (12.3%)	3 (0.7%)
	MSD Positive	3	163			
Samples from Untreated Individuals (N = 383)^b^						
Anti-RBD	MSD Negative	212	40	340 (88.8%)	40 (10.4%)	3 (0.8%)
	MSD Positive	3	128			
Anti-N	MSD Negative	225	38	342 (89.3%)	38 (9.9%)	3 (0.8%)
	MSD Positive	3	117			
Samples from Individuals After Bamlanivimab Infusion (N = 72)^c^						
Anti-RBD	MSD Negative	0	0	72 (100%)	0 (0%)	0 (0%)
	MSD Positive	0	72			
Anti-N	MSD Negative	8	18	54 (75.0%)	18 (25.0%)	0 (0%)
	MSD Positive	0	46			

a Percent concordance calculated as number of samples that tested negative by both Bio-Plex and MSD + number of samples that tested positive by both Bio-Plex and MSD as a proportion of total number of samples tested by both assays.

b Samples include 700 mg and 7,000 mg placebo at all time points and 700 mg and 7,000 mg bamlanivimab arm samples at baseline only (collected prior to infusion).

c Samples include follow-up bamlanivimab-infused time points only (700 mg and 7,000 mg collected at day 28 and week 12).

A secondary analysis censoring individuals treated with bamlanivimab (since they would be artificially positive for anti-RBD IgG) showed a similar pattern of concordance between Bio-Plex and MSD to the full analysis (87% vs 90.5% concordance between assays for anti-RBD for untreated vs all samples, respectively; 86% vs 87% concordance between assays for anti-N for untreated vs all samples, respectively). As expected, all samples collected post-bamlanivimab infusion were positive for anti-RBD IgG by both assays, but 25% negative by MSD were positive by Bio-Plex for anti-N IgG, consistent with the greater sensitivity for anti-N IgG by Bio-Plex (100% vs 93.8% for MSD; Table 1).

**Figure 1.**
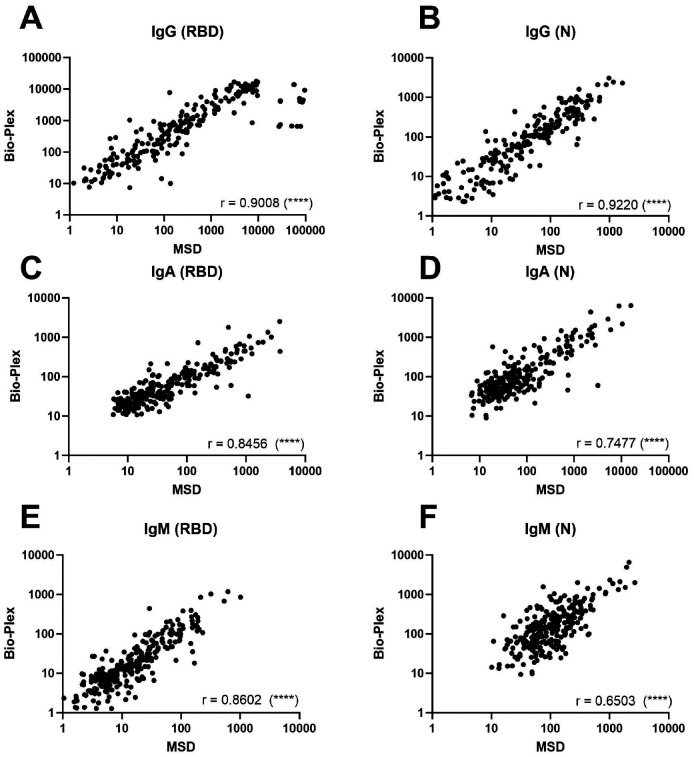
**Comparison of total IgG, IgM, and IgA antibody response to the SARS-CoV-2 RBD and N proteins using Bio-Plex and Meso Scale assays in ACTIV-2/A5401 participants at study entry (pre-treatment, within 10 days of COVID-19 symptom onset).** For all graphs, log Binding Antibody Units per milliliter of serum (BAU/mL) were plotted for Bio-Plex (y-axis) against Meso Scale (x-axis). For both assays, BAU/mL was calculated by applying the conversion factor to the concentration values for each assay; only values with detectable MFI or signal in both assays are plotted on this graph. Paired values where one or both MFI or signal were undetectable and did not produce a quantifiable MFI or signal value are excluded from the correlation. The Spearman Rank Correlation was calculated for each Bio-Plex/MSD pair using GraphPad Prism version 9.5.0 with R value noted in each graph. The P-value for all xy pairs was < 0.0001, denoted by (****) on each graph. (A) Anti-RBD IgG, N = 222 pairs; (B) Anti-N IgG, N = 220 pairs; (C) Anti-RBD IgA, N = 267 pairs; (D) Anti-N IgA, N = 239 pairs; (E) Anti-RBD IgM, N = 268 pairs; and (F) anti-N IgM, N = 268 pairs.

**Comparison of quantitative total IgG, IgM, and IgA antibody response to the SARS-CoV-2 RBD and N proteins using Bio-Plex and Meso Scale assays.** Total IgG, IgM, and IgA antibody levels were assayed in sera by both the Bio-Plex and MSD platforms for 304 participants with COVID-19 in ACTIV-2/A5401 at entry (pre-treatment). Antibody levels converted to the WHO standard BAU/mL were significantly correlated for all isotypes (IgG, IgM, and IgA) and SARSCoV-2 antigen targets (RBD and N) tested that were common between the two assays, with Spearman r values ranging from 0.65 to 0.92 (*P* < 0.0001 for all) (Figure 1).

**Figure 2.**
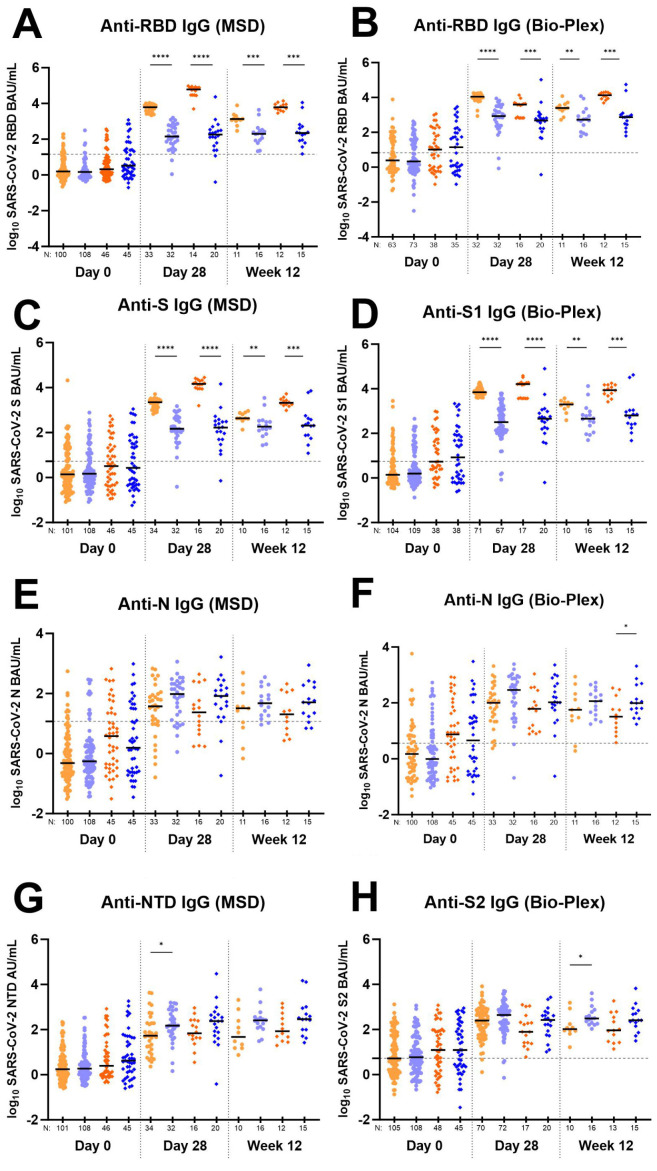
**Total antibody response to various SARS-CoV-2 antigens over time in individuals receiving bamlanivimab and placebo.** Data generated using the Meso Scale Discovery V-PLEX COVID-19 Panel 1 serology assay (MSD) are in panels (A), (C), (E), and (G). Data generated using the Bio-Rad Bio-Plex Pro Human SARS-CoV-2 serology assay are in panels (B), (D), (F), and (H). For all graphs, log_10_ Binding Antibody Units per milliliter of serum (BAU/mL) were plotted for each A5401 study arm as follows: 700 mg bamlanivimab (light orange circles 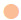); 700 mg placebo (light blue circles 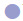); 7,000 mg bamlanivimab (dark orange diamonds 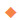); 7,000 mg placebo (dark blue diamonds 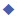). Each arm is plotted from samples collected at baseline (day 0, within 10 days of symptom onset), day 28, and week 12 post-enrollment. For Bio-Plex, seronegative samples whose BAU/mL was under the limit of quantitation could not be plotted thus were omitted from the graph. The median log BAU/mL for each data set is indicated by a solid line. The number of samples plotted is noted under the x-axis. The dashed horizontal line from the y-axis denotes the log BAU/mL value under which the sample is classified as IgG negative; these values are set at (A) 1.17 (anti-RBD IgG MSD); (B) 0.83 (anti-RBD IgG Bio-Plex); (C) 1.25 (anti-S IgG MSD); (D) 0.74 (anti-S1 IgG Bio-Plex); (E) 1.07 (anti-N IgG MSD); (F) 0.56 (anti-N IgG Bio-Plex); (G) no qualitative cutoff is available for anti-NTD IgG MSD; and (H) 0.72 (anti-S2 IgG Bio-Plex). Mann-Whitney test was used to compare unpaired ranked log BAU/mL for each arm (700 mg or 7,000 mg bamlanivimab) against its corresponding placebo arm for each time point of collection. Comparisons are denoted as follows: not significant (ns); *P* ≤ 0.05 (*); *P* ≤ 0.01 (**); *P* ≤ 0.001 (***) and *P* ≤ 0.0001 (****).

**Total antibody response to SARS-CoV-2 Receptor Binding Domain (RBD) antigen over time in individuals receiving bamlanivimab or placebo.** Antibody levels against RBD at entry (day 0), day 28, and week 12 were compared in individuals receiving 700 mg or 7,000 mg placebo or 700 mg or 7,000 mg bamlanivimab to prevent severe COVID-19 using both the MSD (Figure 2A) and Bio-Plex (Figure 2B) assays.

Anti-RBD IgG levels were not significantly different at baseline between intervention arms (bamlanivimab vs placebo) as determined by both MSD and Bio-Plex. On day 28, median anti-RBD IgG levels were significantly higher than placebo (P ≤ 0.001) for both the 700 mg and 7,000 mg arms by both assays. Further, median anti-RBD IgG levels for the 7,000 mg bamlanivimab arm were significantly higher than the 700 mg bamlanivimab arm (*P* ≤ 0.0001), likely reflecting the detection of the mAb, which targets RBD, in the serum (Figures 2A and 2B). Similar results were noted for anti-S IgG (MSD) and anti-S1 IgG (Bio-Plex) as expected, since bamlanivimab targets an epitope on the spike protein of SARS-CoV-2 (Figure 2C and 2D) [27].

In both assays, the median IgG levels between participants who received the mAb or placebo remained significantly different at week 12 for both the 700 mg (*P* ≤ 0.05) and 7,000 mg (*P* ≤ 0.0001) arms; however, the level of anti-RBD IgG for the active arms declined by week 12, likely reflecting a decline in bamlanivimab concentrations, while the IgG levels for the placebo arms remained the same (Figure 2A and 2B).

**Total antibody response to SARS-CoV-2 Nucleocapsid (N) antigen over time in individuals receiving bamlanivimab or placebo.** Since mAb treatment leads to more rapid viral decay, we investigated whether individuals receiving bamlanivimab may have lower levels of host antibody response at day 28. While day 28 and week 12 anti-RBD, anti-S, and anti-S1 antibody levels in the treatment arms reflect both host and therapeutic antibody concentrations, levels of anti-N, anti-NTD, and anti-S2 IgG solely represent the host immune response. Therefore, we further explored anti-N, anti-NTD, and anti-S2 IgG response between those receiving mAb and placebo. We found a consistent trend in the data showing higher levels of host-derived IgG antibodies in placebo compared to treatment arms for both day 28 and week 12 time points (Figures 2E, 2F, 2G, 2H). Four participants from the 700 mg treatment arm developed outgrowth of virus due to monoclonal antibody resistance, as described previously [28]. Since these participants experienced an unusual viral rebound during treatment that we reasoned could influence antibody responses, we conducted an additional analysis of the data without inclusion of these participants. The differences in host-derived IgG antibodies in placebo compared to treatment arms reached statistical significance for day 28 anti-N (MSD and Bio-Plex) and anti-NTD (MSD) (Table 3).

**Table 3.** SARS-CoV-2 IgG Levels in Individuals with No Emergent Bamlanivimab Resistance in the 700 mg Bamlanivimab Arm Compared to Individuals in the 700 mg Placebo Arm in the ACTIV-2/A5401 Study

SARS-CoV-2 Antibody	Day	700 mg Placebo		700 mg Bamlanivimab, No Emergent Resistance		700 mg Bamlanivimab, Emergent Resistance	
		Log_10_ Median (Min, Max) BAU/mL^a^	N	Log_10_ Median (Min, Max) BAU/mL^a^	N	Log_10_ Median (Min, Max) BAU/mL^a^	N
Anti-N IgG (MSD)	0	−0.26 (-1.43, 2.47)	106	−0.33 (-1.51, 2.74)	97	−0.29 (-0.61, 0.35)	3
	28	1.98 (0.055, 3.06)	32	1.55 (-0.79, 2.84)*	29	2.18 (1.07, 2.80)	4
Anti-NTD IgG (MSD)	0	0.27 (-0.49, 2.53)	108	0.26 (-0.60, 2.33)	98	-0.21 (-0.49, 0.0044)*	3
	28	2.18 (0.16, 3.19)	32	1.68 (0.36, 3.64)*	30	2.09 (1.72, 2.57)	4
Anti-N IgG (BioPlex)	0	−0.01 (-1.03, 2.73)	69	0.17 (-1.33, 3.76)	60	No data	0
	28	2.46 (-0.68, 3.39)	32	2.00 (0.37, 2.99)*	27	2.46 (1.67, 3.32)	4
Anti-S2 IgG (BioPlex)	0	0.77 (-0.67, 3.06)	108	0.78 (-0.88, 3.11)	102	0.11 (0.050, 0.72)	3
	28	2.64 (-0.14, 3.71)	72	2.39 (0.11, 3.92)	66	2.46 (1.66, 3.24)	4

a The Mann-Whitney test for a 2-group non-parametric comparison from version 10.1.2 of the GraphPad Software was used to calculate statistical significance between the median log BAU/mL of the 700 mg placebo arm and the 700 mg bamlanivumab arm in individuals with no bamlanivimab-emergent resistance in SARS-CoV-2 and separately in individuals with bamlanivimab-emergent resistance in SARS-CoV-2.

* Significance at *P* < 0.05 is indicated by **bold.**

**Figure 3.**
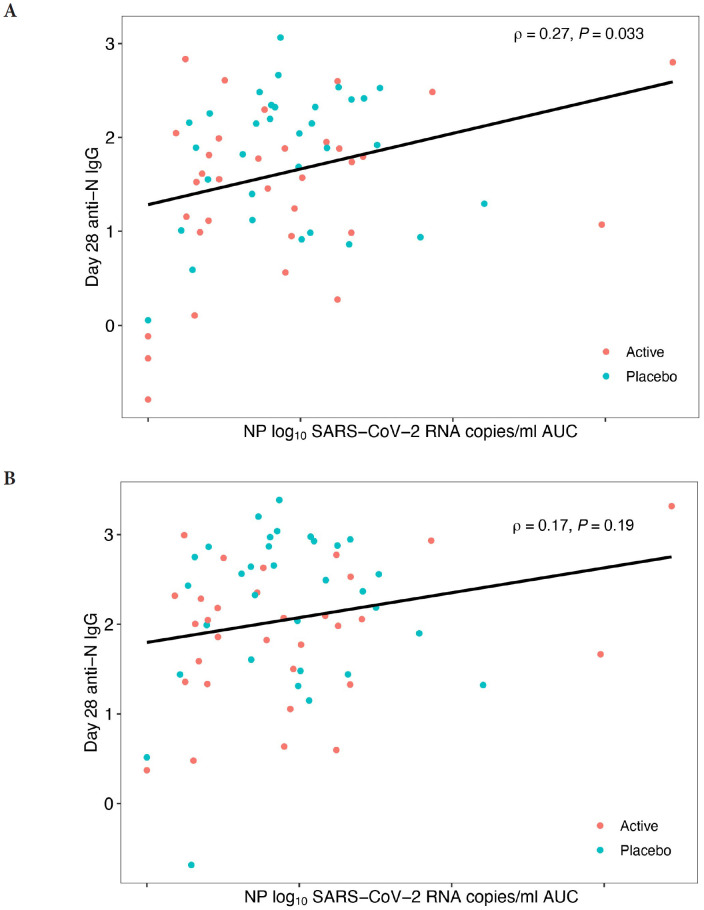
**Area Under the Curve (AUC) of Day 28 Anti-N IgG against SARS-CoV-2 Viral Load.** Blue dots represent 700 mg placebo arm samples, and orange dots represent 700 mg bamlanivimab samples. Data generated using the Meso Scale Discovery V-PLEX COVID-19 Panel 1 serology assay (MSD) in (A) and the Bio-Rad Bio-Plex Pro Human SARS-CoV-2 serology assay in (B). X-axis shows nasoparyngeal (NP) log_10_ SARS-CoV-2 RNA copies/ml area under curve (AUC). Only *P*-values < 0.05 are considered significant and are denoted by P, while Spearman's rho is indicated by ρ.

The tendency for reduced host antibody levels in treated arms versus placebo might be explained by the decreased SARS-CoV-2 RNA levels after mAb therapy leading to reduced antigenic stimulation of the host immune response [23], a concept that is supported by the observation that area-under-the-curve of viral RNA levels days 0 to 28 was positively correlated with day 28 anti-N IgG levels in both the Bio-Plex and MSD assays, reaching statistical significance for the MSD assay (Figure 3). Additionally, assessment of immune responses in the 4 individuals treated with the 700 mg of bamlanivimab who were found to have emerging mAb resistance demonstrated a stronger anti-N IgG response (MSD) at day 28 (median 2.18 log BAU/mL) compared to participants treated with bamlanivimab who did not develop resistance (median 1.55 log BAU/mL). This is likely related to evidence of viral rebound and increased antigen exposure after development of resistance mutations [28]. A similar trend was observed using Bio-Plex.

## DISCUSSION

Although many commercial assays have been developed for the clinical diagnosis of SARS-CoV-2 infection, the advantage of research-use assays for COVID-19 vaccine and prophylaxis studies is that antibody titer can be quantified and differentiated for different isotypes (IgG, IgA, and IgM) as well as different targets (RBD, N, NTD, S1, S2, total S). Research-use assays may be particularly advantageous for studies of mAb against RBD, where detection of host antibody response may be masked in commercial kits by the presence of the mAb. However, differences in kit characteristics and lack of gold standards for assay harmonization may yield discrepant results from the same samples. Our study is the first to use a large set of samples from a COVID-19 mAb clinical trial to compare the qualitative and quantitative performance of two research-use assays: the MSD V-PLEX COVID-19 Panel 1 serology assay and the Bio-Rad Bio-Plex Pro Human SARS-CoV-2 serology assay.

Using serum collected from this trial in non-hospitalized adults with COVID-19 who received the mAb, bamlanivimab, or placebo, our findings indicate that results from these two methodologies are largely concordant when comparing quantitative (titers) measures. However, we observed 10% discordance in qualitative interpretation (serostatus) between MSD and Bio-Plex for RBD and a 13% discordance in interpretation for N. Other antigens could not be evaluated due to lack of common targets between the two assays. These data serve to cross-validate the methodologies but also highlight limitations in serostatus determination that appears to be at least partly explained by imperfection in defining thresholds. Assay thresholds for qualitative interpretation were provided by the manufacturer who set the threshold after testing several hundred pre-COVID specimens at a specific dilution or determined in-house. A limitation of laboratory-determined thresholds is that they may lack specificity due to the impracticality of testing hundreds of pre-COVID specimens collected from separate individuals at multiple dilutions, therefore increasing the rate of discordant results between the two assays. The use of a third assay could serve as a tiebreaker to identify false negatives and false positives, but not without limitations, as the third assay would be subject to its own seropositive thresholds, specificity, and sensitivity. Measuring multiple isotypes may also be a strategy to increase sensitivity; in one study, measuring IgM and IgA antibodies to RBD along with IgG resulted in a 9% increase in identification of positive cases [29].

By contrast, we observed a strong correlation (*P* < 0.0001) between MSD and Bio-Plex for quantitative antibody response to RBD and N for all three isotypes (IgG, IgA, IgM). Specific quantification of antibodies against different S and N proteins and their subunits can aid in quantifying host response to infection since many vaccines and prophylactic monoclonal antibodies target regions in S [30]. A challenge of comparing assay results quantitatively was that each manufacturer had its own units of measure, typically denoted as “arbitrary” units. The WHO recently set the first International Standard for humoral immune response assessment, prompting manufacturers to provide a conversion factor to report results in Binding Antibody Units (BAU). In our study, we were able to convert all antibody titers to BAU/mL, except for MSD anti-NTD IgG, due to a lack of conversion factor for that target. When evaluating the findings from the ACTIV-2/A5401 study, we observed that both assays produced similar trends in antibody activity, but the actual titers differed by assay when comparing values using the same units (BAU/mL). Thirteen outliers were observed in Figure 1A at the highest MSD titers, which could, hypothetically, reflect differences in the MSD and Bio-Plex in detecting the monoclonal antibody itself.

The ACTIV-2/A5401 study demonstrated an important application of the MSD and Bio-Plex assays in quantitatively assessing host versus therapeutic antibody levels. Each assay detected different antigens (anti-N and anti-S2 by Bio-Rad and anti-N and anti-NTD by MSD), allowing detailed assessment of host immune response between study arms.

We found that for both Bio-Rad and MSD platforms, IgG antibody levels for host immune responses (as indicated by anti-N, anti-NTD, and anti-S2 titers) all showed numerically lower levels in the bamlanivimab-treated arms compared to the placebo. We propose that this may be a consequence of reduced overall exposure to viral antigens with monoclonal antibody treatment. This concept is also supported by the observations that viral load as measured by AUC was positively associated anti-N antibody titers. Also, 4 participants with resistant virus displayed anecdotal evidence of increased anti-N IgG responses compared to participants without emergence of resistant virus. A similar analysis could not be done for the 7,000 mg arm since there were no individuals with emergent resistance in this group.

Evidence of diminished host antibody responses to COVID-19 in patients receiving monoclonal antibody therapy during acute infection have also been described by others [31] and similar to our observations, found evidence of a relationship between viral load and antibody responses. Although host antibody responses may be diminished by early administration of monoclonal antibodies, it is important to recognize that host antibody responses are still readily detectable in these circumstances, and the clinical significance of slightly reduced titers is unclear.

In conclusion, SARS-CoV-2 antibody assays provide essential data for characterizing the immune responses with and without treatment in a study population and for allowing subgroup analyses of treatment effects by serostatus. The benefit of both the MSD and Bio-Plex assays is the ability to quantify antibody response and differentiate between antigens and isotypes. Future studies could use either or both platforms, which would give congruent results to look for trends in antibody response; however, research-use assays cannot be used for individual diagnosis, and the advantage of research assays is the ability to quantify antibody response to specific antigens rather than to classify samples as positive or negative. Different platforms yielding some degree of discordance is expected for serology assays, especially when using serum samples with low antibody concentrations, as can be expected early in the course of infection [32, 33]. Whereas some investigators have suggested that studies could benefit from inclusion of multiple serology assays, the cost of such an approach may be prohibitive. In this study, we used a small validation study comparing two platforms, with results that were largely reflective of those observed in clinical trial samples. Thus, small validation studies with different serology platforms may be a more practical approach for clinical trial design.

## AUTHOR STATEMENTS

U.M.P. and S.F.S. designed the study and wrote the manuscript. A.L.H., D.M., K.C.G., M.C.C., R.D., and C.M. conducted experiments and performed analysis. J.W.M., P.K., A.L.L., J.S.C., J.J.E., K.W.C., D.M.S., and J.Z.L. contributed to study interpretation and manuscript review. K.W.C. and D.M.S. conducted the study from which samples were obtained.
